# The Role of AP-1 in Cancer: Regulation, Tumor Microenvironment and Therapeutic Targeting

**DOI:** 10.3390/biom16071048

**Published:** 2026-07-17

**Authors:** Maria A. Katsianou, Dimitrios Vrachas, Christos Adamopoulos

**Affiliations:** 1 Department of Biological Chemistry, Medical School, National and Kapodistrian University of Athens, 11527 Athens, Greece; makatsianou@med.uoa.gr; 2 Department of Oncological Sciences, Icahn School of Medicine at Mount Sinai, New York, NY 10029, USA; dimitrisvra@med.uoa.gr

**Keywords:** AP-1, tumorigenesis, MAPK pathway, targeted therapy, tumor microenvironment

## Abstract

The activator protein-1 (AP-1) transcription factor is a regulatory dimeric transcription factor complex, that responds to a wide range of intracellular and extracellular stimuli and controls gene expression involved in tumor initiation and progression. Comprised primarily of members of Jun and Fos protein subfamilies, AP-1 is activated downstream of major oncogenic signaling pathways such as the mitogen-activated protein kinase (MAPK) pathway and controls cellular processes including differentiation, invasion, proliferation and apoptosis. In various cancer types, AP-1 contributes to tumor growth by promoting tumor-like phenotypes and facilitating metastatic behavior. Furthermore, AP-1 can affect the tumor microenvironment by modulating inflammation and interaction with immune cells. AP-1 deregulation is linked to tumor heterogeneity and resistance to chemotherapy and radiation. Therefore, AP-1 has emerged as a potential therapeutic target. In preclinical models, direct and indirect targeting via upstream pathways of AP-1 components has demonstrated encouraging results. In addition, combinatorial approaches targeting AP-1 and other regulators may improve the effectiveness of treatment and overcome therapy resistance. In this review, we highlight the AP-1’s role as a critical hub in tumorigenesis that links oncogenic signaling to transcriptional regulation. We also focus on its regulation, function in the tumor microenvironment, and therapeutic potential in combating tumors.

## 1. Introduction

**Figure 1:**
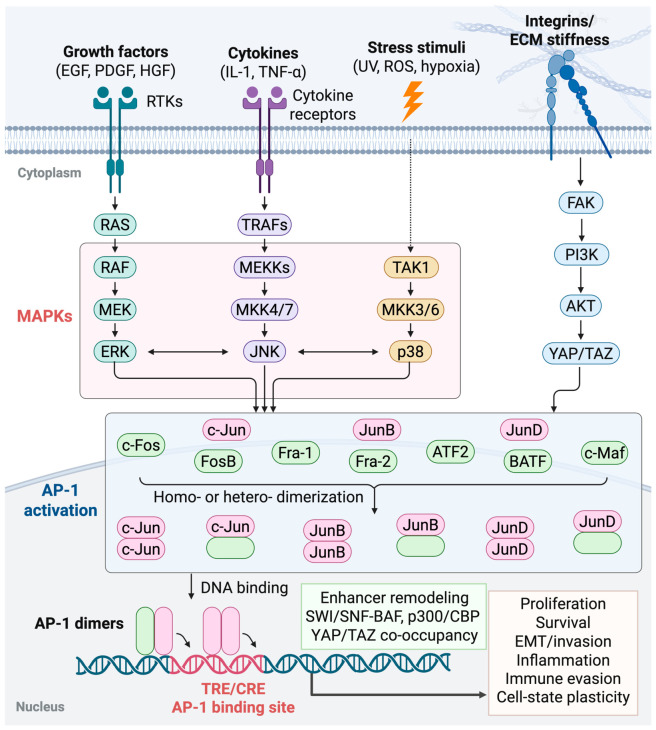
Signaling pathways regulating AP-1 activation and transcriptional output in cancer. AP-1 acts as a signal-responsive transcription factor complex that integrates oncogenic, inflammatory, stress-related, and mechanotransduction cues. Growth factors, cytokines, cellular stress, hypoxia, ROS, ECM stiffness, and integrin signaling converge mainly through MAPK/ERK, MAPK/JNK, MAPK/p38, FAK–PI3K–AKT, and YAP/TAZ-associated pathways to regulate the expression, stability, phosphorylation, and activity of AP-1 components, including Jun, Fos, ATF, BATF, and Maf family proteins. AP-1 dimers bind TRE/CRE regulatory elements and cooperate with chromatin and enhancer-remodeling factors, such as SWI/SNF–BAF and p300/CBP, to control transcriptional programs involved in proliferation, survival, EMT and invasion, inflammation, immune evasion, and cell-state plasticity. AKT, protein kinase B; AP-1, activator protein-1; ATF2, activating transcription factor 2; BATF, basic leucine zipper ATF-like transcription factor; CRE, cAMP-responsive element; ECM, extracellular matrix; EGF, epidermal growth factor; EMT, epithelial–mesenchymal transition; ERK, extracellular signal-regulated kinase; FAK, focal adhesion kinase; HGF, hepatocyte growth factor; IL-1, interleukin-1; JNK, c-Jun N-terminal kinase; MAPK, mitogen-activated protein kinase; MEK, mitogen-activated protein kinase kinase; MEKK, mitogen-activated protein kinase kinase kinase; MKK, mitogen-activated protein kinase kinase; PDGF, platelet-derived growth factor; PI3K, phosphatidylinositol 3-kinase; RAF, rapidly accelerated fibrosarcoma kinase; RAS, rat sarcoma viral oncogene homolog; ROS, reactive oxygen species; RTK, receptor tyrosine kinase; SWI/SNF–BAF, switch/sucrose non-fermentable-BRG1/BRM-associated factor chromatin-remodeling complex; TAK1, transforming growth factor-β-activated kinase 1; TAZ, transcriptional coactivator with PDZ-binding motif; TNF-α, tumor necrosis factor-α; TRAF, tumor necrosis factor receptor-associated factor; TRE, TPA-response element; UV, ultraviolet; YAP, Yes-associated protein. Created in BioRender. Adamopoulos, C. (2026) https://BioRender.com/9snq84c (accessed on 7 July 2026).

AP-1 is a transcription factor complex formed by hetero- or homo-dimers from members of the activating transcription factor (ATF), Fos (c-Fos, FosB), Fos-related antigen (Fra-1/FOSL1, Fra-2/FOSL2), Jun (c-Jun, JunB, JunD), and musculoaponeurotic fibrosarcoma oncogene family (MAF) subfamilies, which are members of the basic region-leucine ZIPper (bZIP) class of DNA-binding proteins [1]. Dimerization enables AP-1 to orchestrate transcription, depending on cell type and context [2]. Dimer composition defines AP-1 signaling outcome, as c-Fos/c-Jun heterodimers are often related to oncogenesis while JunB and JunD are associated with tumor-suppressive transcriptional programs (Figure 1).

Notably, AP-1 should not be considered solely oncogenic. Its function is highly context dependent and is defined by various parameters such as dimerization, chromatin accessibility, tissue type and cellular environment, allowing AP-1 to exert either oncogenic or tumor suppressive functions [3].

Single-cell and multi-omics, including Assay for Transposase-Accessible Chromatin sequencing (ATAC-seq) and Chromatin Immunoprecipitation sequencing (ChIP-seq) have revealed that AP-1 binding motifs exhibit cell-type specificity and dynamic regulation [2]. AP-1’s binding to specific motifs enables various upstream signals to transcriptionally regulate genes involved in proliferation, survival and stress resistance. AP-1 can integrate signals from cyclic adenosine monophosphate (cAMP)-dependent pathways by binding to DNA consensus sequences, including the cAMP-responsive element (CRE, 5′-TGAGCGTCA-3′) and the palindromic 12-O-tetradecanoylphorbol-13-acetate (TPA)-response element (TRE, 5′-TGAG/CTCA-3′) [4]. Beyond direct binding, AP-1 facilitates promoter–enhancer interactions by cooperating with other chromatin regulators and transcription factors, and thus controls enhancer activity and gene expression (Figure 1) [5].

Various extracellular (e.g., growth factors, proinflammatory cytokines, interferons, bacterial toxins, viral infections, mechanical strain or shear stress, as well as UV and ionizing radiation) or intracellular stimuli (e.g., oxidative and glycative stress or DNA damage), can activate AP-1 [6]. In both cases, AP-1 activation is primarily mediated through the activation of certain intracellular signaling pathways such as the mitogen-activated protein kinase (MAPK) and phosphatidylinositol 3-kinase/protein kinase B (PI3K/Akt) signaling pathways (Figure 1) [2,7,8,9]. In cancer cells, particularly, AP-1-activation is driven largely by the oncogenic rat sarcoma (RAS)-MAPK signaling pathways as well as the associated post-translational modifications such as phosphorylation, ubiquitination and SUMOylation [10].

AP-1 is implicated in the pathobiology of a variety of diseases, such as cancer and immune system disorders, such as rheumatoid arthritis, psoriasis and systemic lupus erythematosus [11]. Among them, cancer is the most extensively studied, as tumor progression, cell transformation, aggressiveness and therapy resistance are evidently correlated with dysfunctional AP-1 [12]. Elevated levels of AP-1 have been found in many tumors, including anaplastic large cell lymphoma, triple-negative breast cancer (TNBC), colorectal cancer, melanoma and Hodgkin’s lymphoma [13,14,15]. High expression of specific AP-1 components, notably JunB has been associated with poor prognosis and reduced survival [16]. AP-1 is also implicated in the shaping of the tumor microenvironment, enhancing epithelial–mesenchymal transition (EMT) and cancer cell plasticity [17].

Due to its crucial role, AP-1 as well as its upstream regulators and downstream effectors, represent therapeutic targets and therefore are the subject of several analytical studies [7]. Although AP-1 targeting remains challenging due to its complex structure, recent studies focus on targeting upstream regulators such as MAPKs or act at an epigenetic level. Also, AP-1-inhibition in combination with other therapies is assessed. Notably, AP-1 is a key mediator of drug resistance development, specifically in Epidermal growth factor receptor (EGFR)-targeting therapies. Thus, novel therapeutic schemes including rational combinatorial strategies as well as multi-omics-guided targeting should be further explored, to overcome resistance limitations [12].

Although the oncogenic functions of AP-1 have been summarized in a plethora of comprehensive reviews, recent studies have expanded our knowledge on how AP-1 regulation depends on context, ultimately influencing therapy. More specifically, single-cell and multi-omics technologies have revealed that AP-1 binding or dimerization happens to specific cell types. Furthermore, emerging evidence has elucidated the role of AP-1 in tumor plasticity and tumor microenvironment as well as resistance to targeted therapies. This review integrates these recent developments along with a detailed overview of current and emerging therapeutic strategies targeting AP-1 signaling including upstream pathway inhibition, epigenetic alteration and combination therapies, with the aim of analyzing the potential of AP-1 translation in cancer. We further summarize the key mechanisms regulating AP-1 activity, its role in tumorigenesis and tumor progression, and the current therapeutic AP-1 targeting approaches.

## 2. AP-1 Regulation

The MAPK signaling pathways are the principal regulators of AP-1 activity and control fundamental cellular functions such as proliferation, apoptosis, survival, and differentiation in response to a variety of stimuli including cytokines, oncogenes, and growth factors [4,7,18]. Activation of the MAPK pathways occurs through a sequence of phosphorylation events targeting serine/threonine sites on specific cytoplasmic kinases, namely MAPK kinase kinases (MAP3Ks), such as B-raf proto-oncogene serine/threonine-protein kinase (BRAF) or apoptosis signal-regulating kinase 1 (ASK1), and MAPK kinases (MAP2Ks), such as mitogen-activated protein kinase kinase 1/2 (MEK1/2) or stress-activated protein kinase/ERK kinase 1 (SEK1). These signaling pathways lead to the phosphorylation and activation of the terminal MAPKs, including extracellular signal-regulated kinase 1/2 (ERK1/2), c-Jun N-terminal kinase (JNK) and p38 kinase (Figure 1) [19].

Once activated, ERK1/2 phosphorylates transcription factors such as ETS Like-1 protein Elk-1 (Elk-1), a member of the TCF (ternary complex factor) family. Elk-1 binds to the *Fos* promoter, which induces the formation of active Fos and Jun heterodimers, which comprise the active AP-1 complexes [7,20]. ERK1/2 also enhances *JunB* transcription by activating Ets-1, an ETS-domain transcription factor that binds directly to the *JunB* promoter, amplifying the AP-1 expression [7]. JNK phosphorylates c-Jun, at Ser63 and Ser73, and ATF-2 within its N-terminal activation domain, at Thr63 and Thr71, inducing their transcriptional activity. p38 MAPK also phosphorylates ATF-2 at Thr69 and Thr71, leading to its activation [7]. AP-1 activity is regulated, as well, by the PI3K/Akt axis. An activated PI3K/Akt signaling enhances AP-1 transcriptional capacity, herein affecting protein stability, nuclear localization, and the recruitment of co-factors which regulate gene expression [21]. Such cell signaling interconnection highlights the role of AP-1 as a downstream target of multiple oncogenic stimuli. Importantly, AP-1 transcription factors, along with lineage-specific transcription factors, contribute to the establishment of an accessible chromatin state by selecting cell-dependent enhancer landscapes, thereby linking extracellular signaling to transcriptional output (Figure 1) [22].

AP-1 components associated with dysregulated MAPK pathway components’ activity can function as either oncogenes or tumor suppressors. In this case, aberrant signaling can trigger expression of different AP-1 target genes, as well as functional activation and stabilization of AP-1 proteins, leading to alterations of oncogenic transcriptional activity (Figure 1) [7].

Importantly, AP-1 regulation also occurs at the post-translational level. Methylation, phosphorylation, ubiquitination and acetylation alter AP-1 components’ stability, influencing protein turnover and transcription [23]. Dysfunctional ubiquitin–proteasome system components maintain AP-1 signaling in cancer by preventing Jun and Fos protein turnover, thereby prolonging oncogenic transcriptional responses [24].

AP-1 is also regulated by epigenetic mechanisms and non-coding RNAs. AP-1 is recruited to regulatory elements, a recruitment defined by accessible chromatin, DNA methylation, histone marks and ATP-chromatin-remodeling complexes [22]. This alters the transcription of AP-1 target genes in a cell type-specific manner, particularly through cooperation with chromatin remodelers such as the BAF complex, promoting enhancer accessibility [22]. Non-coding RNAs also regulate gene expression and chromatin remodeling. The lncRNA DUBR regulates AP-1 binding site accessibility through epigenetic mechanisms, controlling transcription involved in cancer cell survival and differentiation [25].

Furthermore, AP-1, along with Yes-associated protein (YAP)-Transcriptional coactivator with PDZ-binding motif (TAZ), and transcriptional regulators of the Hippo pathway, which regulate genes responsible for S-phase entry and cell mitosis, can co-occupy chromatin [26]. Such an association complex promotes skin tumorigenesis, leading to melanoma development [27]. AP-1 inhibitors, as well as upstream and downstream targeting molecules, are being assessed in several tumors like respiratory epithelium cancers, lung cancer and lymphomas [28,29]. AP-1 can display pioneer transcription factor (PTF) properties in cooperation with chromatin remodeling complexes such as the mammalian switch/sucrose non-fermentable (SWI/SNF) complex, or BRG1/BRM-associated factor (BAF) complex [30]. In addition, the formation of transcriptional complexes at active regulatory regions is enabled by AP-1, which in turn facilitates the binding of co-activators such as adenovirus early region 1A (E1A), binding protein p300/CREB-binding protein (p300/CBP) and RNA polymerase II [31,32]. Collectively, AP-1 plays a key role in oncogenic signaling, where mechanisms like kinase signaling and chromatin remodeling define transcription (Figure 1).

## 3. AP-1 in Cancer

### 3.1. Breast Cancer

**Figure 2:**
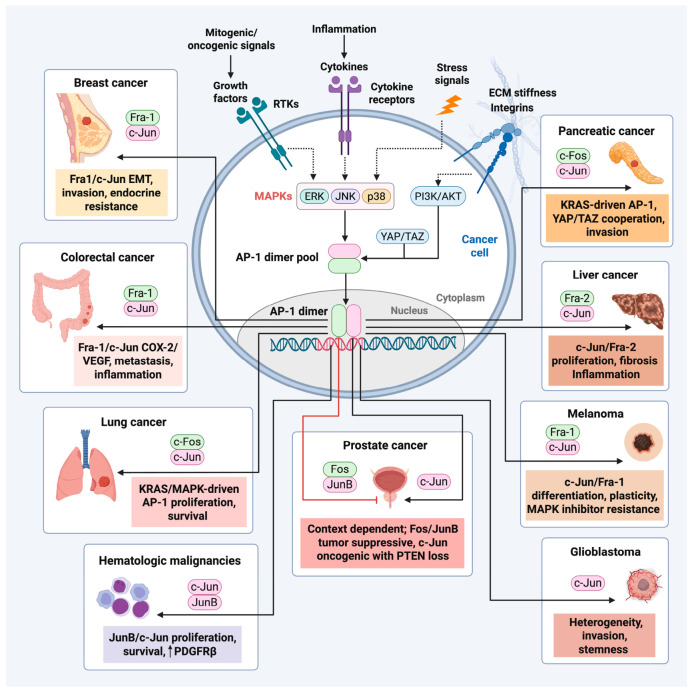
Context-dependent roles and functional properties of AP-1 across cancer types. Mitogenic and oncogenic signals, inflammatory cytokines, cellular stress, and ECM stiffness/integrin signaling converge on MAPK, PI3K/AKT, and YAP/TAZ-associated pathways to regulate the AP-1 dimer pool and DNA-bound AP-1 transcriptional complexes in cancer cells. Depending on AP-1 subunit composition and tumor context, AP-1 drives distinct transcriptional programs across malignancies. In breast cancer, colorectal cancer, lung cancer, pancreatic cancer, liver cancer, melanoma, hematologic malignancies, and glioblastoma, AP-1 components promote cancer-associated processes including proliferation, survival, EMT, invasion, inflammation, metastasis, plasticity, stemness, and therapy resistance. In prostate cancer, AP-1 function is particularly context-dependent, with Fos and JunB displaying tumor-suppressive roles, whereas c-Jun may exert oncogenic activity in the setting of PTEN loss. AP-1, activator protein-1; AKT, protein kinase B; COX-2, cyclooxygenase-2; ECM, extracellular matrix; EMT, epithelial–mesenchymal transition; ERK, extracellular signal-regulated kinase; JNK, c-Jun N-terminal kinase; MAPK, mitogen-activated protein kinase; PDGFRβ, platelet-derived growth factor receptor beta; PI3K, phosphatidylinositol 3-kinase; PTEN, phosphatase and tensin homolog; RTK, receptor tyrosine kinase; VEGF, vascular endothelial growth factor; YAP/TAZ, Yes-associated protein/transcriptional coactivator with PDZ-binding motif.

Oncogenic signaling, hormone receptor activity and chromatin remodeling merge with AP-1 to drive breast tumorigenesis (Figure 2). Genomic analyses link AP-1 to enhancer remodeling and transcriptional reprogramming in breast cancer [33]. Furthermore AP-1 plays a key role in both tumor progression and therapeutic response [28,29,34,35]. More specifically, several of its subunits, including c-Jun, Fra-1 and Fra-2, are overexpressed in breast tumors. Fra-1 is overexpressed in TNBC, with differential expression between estrogen receptor α (ERα) positive and ERα negative breast cancers [34]. In vitro studies have demonstrated that Fra-1 expression is associated with increased cell motility, proliferation and invasiveness [35,36]. Furthermore, elevated Fra-1 expression in both TNBC cell lines and primary tumor samples is linked to poor clinical prognosis [36]. However, Fra-1 expression does not consistently correlate with nodal or metastatic status and its prognostic value remains uncertain [36]. Elevated *Fra-2* mRNA levels have been associated with younger patient age, nodal involvement, higher tumor grade and ER negativity [35]. Contrastingly, higher levels of JunB mRNA are associated with reduced tumor size, lower stage and node-negative status, suggesting that JunB affects breast cancer suppressively [34].

At a mechanistic level, AP-1 regulates zinc finger E-box-binding homeobox 2 (ZEB2), a transcriptional repressor of E-cadherin, which in turn facilitates cell invasion and epithelial-to-mesenchymal transition (EMT) in TNBC cells [9]. In addition, AP-1 components such as c-Fos, Fra-1, c-Jun, and JunB, in cooperation with Smad2/3 trigger Transforming growth factor beta (TGFβ)-induced invasion. Smad2/3–Fra1 complexes play a central role in activating invasion-associated genes. The induction of TGFβ-Smad-pro-oncogenic functions via AP-1 in HER2^+^ and/or EGFR^+^ breast cancer cell lines is triggered by EGFR activation and expression of the ΔN isoform of the transcription factor p63. These synergistically enhance the expression of invasion-and migration-related genes, which create an EGFR/AP-1/p63/TGFβ loop that promotes and supports a pro-oncogenic activity, susceptible to pharmacological inhibition [37]. Further to its invasive and proliferative identity, AP-1 regulates key cell cycle genes such as cyclin D1, which promote breast cancer cell growth. More specifically, the direct AP-1 binding to cyclin D1 promoter inhibits cyclin D1 expression, inducing cell cycle arrest [28].

At the chromatin level, studies in mesenchymal TNBC indicate that enhancer permissiveness plays an important role in transcription factor functionality. Chromatin SWI/SNF-mediated remodeling enables activation of the AP-1-associated enhancer to promote TNBC cell progression. Thus, AP-1 transcriptional ability depends on subunit expression and chromatin availability [38].

Hormonal signaling pathways also regulate AP-1. For example, progestins increase the AP-1 transcriptional potential and the recruitment of AP-1 complexes with progesterone receptors to the cyclin D1 promoter, therefore relating hormone signaling to breast cancer proliferation [28,39]. Genome-wide analysis in ER-positive breast cancer cells has proven substantial overlap of sites bound by AP-1 and ER chromatin proteins. Notably, upregulation of c-Jun proteins alters ER binding, regulates ER transcriptional activity, and lowers tamoxifen resistance and this was mediated via TGF-β signaling, revealing a potential route of intervention in ER^+^ tumors [40].

Quantitative binding studies involving chromatin immunoprecipitation combined with synthetic oligonucleotides (ChIP-ISO) in A549 lung cancer cells demonstrated that AP-1 binds through cooperation with the pioneer transcription factor FOΧA1, thus providing the underlying mechanism by which AP-1 acts as part of hormone-dependent transcription through chromatin binding cooperativity [41]. Interestingly, in several human breast cancer cell lines, treatment with the CDK4/6 inhibitor abemaciclib leads to AP-1 activation and increased enhancer binding, affecting transcriptional reprogramming [33].

Further to its direct role in tumor cells, AP-1 may exert context-dependent roles. For example, JunB expressed by stromal cells at metastatic sites may have a tumor-suppressive role by upregulating the expression of certain angiogenic proteins in tumor-infiltrating neutrophils [42]. Single-nucleus RNA and ATAC sequencing of early-stage breast cancer sample treatment revealed that a variability in therapy can remodel gene expression metaprograms as well as cancer cell states, underscoring the importance of the microenvironmental context [43]. Finally, AP-1 composition can influence tumor behavior. In TNBC, cancer stem cells rely on a PKCα-driven shift in AP-1 composition from c-Fos in non-stem cells to Fra1 in stem-like cells, with Fra1 sustaining EMT and stemness properties [44] (Figure 2).

### 3.2. Colorectal Cancer

AP-1 plays a multifaceted role in colorectal carcinogenesis. According to epigenomic studies, increased AP-1 transcriptional activity and function in enhancer regulation are discovered in colorectal cancer (CRC) [45]. Immunohistochemical analyses on human colon adenocarcinoma have shown that AP-1 is present in stromal myofibroblasts surrounding colon tumors. Hence, the expression of AP-1 positively correlates with the expression of downstream targets of AP-1, cyclooxygenase-2 (COX-2) and vascular endothelial growth factor (VEGF) as well as epidermal growth factor receptor (EGFR), which activates AP-1 via MAPK pathways [6]. Studies support that AP-1 remains active at a transcription level in EGFR-driven metastatic CRC, as the deubiquitinase USP21 stabilizes Fra-1 and prevents degradation in metastatic CRC models [46]. Expression of AP1 induces COX-2 expression via the action of either kinase C (PKC) or ERK1/2 signaling. This is associated with the promotion of anti-apoptotic properties, cellular motility and invasion in CRC [47].

Moreover, activation of the Wnt/β-catenin pathway increases expression of AP-1 via direct binding of β-catenin/T cell-factor (TCF)/lymphoid-enhancer-factor (LEF) complex, which is involved in CRC carcinogenesis and metastasis [47]. The involvement of AP-1 is essential for CRC progression, as it determines enhancer landscapes downstream of oncogenic pathways, coexisting with chromatin remodeling proteins [45]. It controls an epigenetic chromatin remodeling program during the colorectal carcinogenesis process [48]. AP-1 associates with the SWI/SNF or BAF chromatin remodeling complex in *KRAS*-mutant CRC cell lines and regulates the transcription of cancer-related genes [48]. Additionally, AP-1 is linked to anti-apoptotic signaling under hypoxic conditions in the solid tumor environment, which provides resistance to chemotherapy and radiotherapy [49].

High AP-1 activity or the overexpression of some of the subunits, specifically Fra-1 and c-Jun, are correlated with poor prognosis and increased metastasis in CRC patients, indicating its clinical and therapeutic target [1]. Both c-Jun and Fra-1 can upregulate mesenchymal markers such as *ZEB1*, *Vimentin*, and *Snail*, and can promote EMT, enhancing the metastatic potential [50]. Specifically, Fra-1 is upregulated in *BRAF*-mutant CRC contexts, sufficient to induce tumorigenesis [51]. AP-1-driven cytokine expression can be further stimulated via interaction with other inflammatory transcription factors, especially nuclear factor kappa B (NF-κB), including interleukin-6 (IL-6) and tumor necrosis factor-α (TNF-α), to reinforce an inflammatory microenvironment that promotes tumor growth [52]. While AP-1 exerts an oncogenic function in CRC, the cellular context may alter its function, as some AP-1 subunits, such as JunD, may instead exhibit tumor-suppressive effects [3] (Figure 2).

### 3.3. Blood Malignancies

AP-1 proteins have been shown to promote proliferation in anaplastic lymphoma kinase (ALK)-positive anaplastic large cell lymphoma (ALCL) and classical Hodgkin lymphoma (cHL) [53]. In the L-428 cHL cell line, inhibition of AP-1 activity through expression of a dominant negative c-Fos construct (A-Fos) resulted in reduced cell proliferation, accompanied by reduced cyclin D2 expression [54]. In addition, pharmacological inhibition of JNK—a key activator of c-Jun—in the SU-DHL-1 ALK-positive ALCL cell line induced G2/M cell-cycle arrest. This effect is probably related to the upregulation of the cyclin-dependent kinase inhibitor p21^Cip1^, and parallel downregulation of cyclin A [54]. Interestingly, a similar cell cycle dysfunction is observed in cHL cell lines following JNK inhibition, which likewise relates to p21^Cip1^ expression [55].

AP-1 proteins’ involvement in proliferation has further been reported through RNA interference approaches such as short interfering RNA (siRNA), short hairpin RNA (shRNA) knock-down experiments, and CRISPR-Cas9 knockout assays. Studies have reported that *JunB* silencing in most ALK-positive ALCL cell lines resulted in decreased proliferation [7,53,56,57]. In contrast, c-Jun exhibits more complex effects on proliferation regulation in ALK-positive ALCL. For instance, siRNA-mediated c-Jun knockdown is associated with reduced cell growth and viability, accompanied by increased p21^Cip1^ expression and decreased levels of cyclins A and D3 [55].

Mechanistically, AP-1 regulates the transcription of genes associated with proliferation and cell survival. In the CD4-NPM-ALK mouse model, in which CD4^+^ T cells express the oncogenic NPM-ALK fusion protein, c-Jun and JunB were shown to drive transcription of platelet-derived growth factor receptor β (PDGFRβ), a receptor tyrosine kinase (RTK) highly expressed in ALK-positive ALCL patient samples and selected ALK-positive ALCL cell lines, where it contributes to tumor growth [58,59].

Furthermore, AP-1 proteins promote proliferation in ALK-positive ALCL cells, through activation of the PI3K/Akt signaling pathway. Studies using BaF3 cells expressing the NPM-ALK fusion protein showed that dominant-negative Akt impaired both colony formation and tumor development [54,59]. McDonnell and colleagues further revealed that NPM-ALK signaling activates the PI3K/Akt pathway, leading to phosphorylation and inactivation of the serine/threonine kinase glycogen synthase kinase 3 β (GSK3β) [60]. In addition, inhibition of GSK3β in ALK-positive ALCL cell lines enhances proliferation by stabilizing Gli, a Sonic Hedgehog (SHH) pathway transcription factor, thereby increasing cyclin D2 expression [61].

In addition to its role in proliferation, AP-1 controls pathways involved in apoptosis and cell survival. Knockdown of either CD30 or Cyp40 proteins is associated with reduced cellular viability, whereas the introduction of a dominant-negative Akt mutant impaired colony formation in BaF3 cells expressing NPM-ALK [54,57,62]. Similarly, both inhibition and knockdown of *MYC* decreased the viability of ALK-positive ALCL cell lines [54,63]. In addition, Akt activation and the subsequent inhibition of GSK3β, prevent phosphorylation and degradation of the pro-survival Bcl-2 family protein Mcl-1 in ALK-positive ALCL [60]. Finally, downstream effectors mammalian target of rapamycin (mTOR) and Forkhead box O3 (FoxO3) are also important for ensuring survival in ALK-positive ALCL [54]. Further studies have confirmed AP-1’s role in the pathogenesis of other types of blood malignancies. For example, in ALCL, the AP-1-basic leucine zipper ATF-like transcription factor (BATF) complex regulates gene expression patterns unique to group 3 innate lymphoid (ILC3) cells and T helper 17 (TH17), by targeting the expression of various marker genes [64].

Similarly to NF-κB, AP-1 is continually activated downstream of the B-cell receptor signaling involving membrane-associated guanylate kinase 1 (CARMA1) and/or the Toll-like receptor adaptor myeloid differentiation primary response gene 88 (MyD88) in activated B-cell-like diffuse large B-cell lymphoma (ABC-DLBCL) cell lines [65].

In multiple myeloma (MM), AP-1—particularly the JunB subunit—is crucial in promoting cell proliferation, survival and drug resistance, highlighting its importance as a promising therapeutic target [66]. Pharmacological inhibition of AP-1 using the selective inhibitor T-5224 has exhibited anti-MM activity in both in vitro and in vivo models [67]. Particularly, T-5224 in MM cells has been shown to induce apoptosis, which can be reversed by treatment with the ferroptosis-specific inhibitor ferropstatin-1 (Fer-1) [68]. Finally, AP-1’s role in hematologic malignancies appears to be highly dependent on cell context, with MYC and JunB displaying only limited overlap in their downstream targets and global chromatin-binding patterns, further underscoring the specificity of AP-1-driven transcriptional regulation [69] (Figure 2).

### 3.4. Respiratory Epithelial and Lung Cancers

Respiratory epithelial carcinogenesis is mediated by complex signaling networks, in which AP-1 functions as a key regulator of proliferation, differentiation and survival. Aberrant AP-1 activity is frequently observed in lung cancer, particularly in non-small cell lung cancer (NSCLC), where it acts downstream of oncogenic signaling pathways such as the KRAS/MAPK signaling axis [70]. Retinoid receptor-mediated inhibition of AP-1 activity exerts a tumor suppressive role, with the retinoic acid receptor (RARα) mediating retinoid antitumor activity in several cancers, including lung cancer [47].

During the early stages of respiratory epithelium carcinogenesis, particularly during the hyperplastic metaplasia phase, carcinogen-induced genetic instability leads to a decreased RARa expression. However, increased AP-1 activity and overexpression of its cofactors promote proliferation, block differentiation and drive malignant transformation [71]. AP-1 regulates the cell cycle primarily through transcriptional activation of cyclin D1, while cyclin D1 degradation contributes to the chemopreventive effects observed in tobacco carcinogen-transformed cells. Additionally, COX-2, an AP-1-dependent gene, is repressed in normal epithelium but upregulated during carcinogenesis, where it contributes to AP-1-mediated survival signaling [47]. In early laryngeal carcinogenesis, elevated levels of c-Fos and phosphorylated/activated c-Jun (p-c-Jun) together with loss of retinoid receptor-induced AP-1, suggest that this regulatory axis acts as a protective mechanism that is lost during malignant progression [72].

AP-1 subunits are critical modulators of lung cancer and exhibit prognostic value. The prognostic effects of the AP-1 transcription factor FosB in NSCLC depend on *TP53* mutational status. Specifically, FosB indicates a positive prognosis in patients with wild-type *TP53* but with a poor prognosis in *TP53*-mutant tumors. AP-1 components are highly context-dependent and influenced by the genetic landscape of the tumor, uncovering FosB as a potential prognostic biomarker and therapeutic target in NSCLC [13].

Also, genome-wide studies suggest that AP-1 regulates chromatin remodeling in lung cancer. AP-1 interacts with chromatin-remodeling complexes, including SWI/SNF (BAF) and promotes transcription of oncogenic genes by establishing and maintaining active enhancer regions [73]. Therefore, AP-1 is involved in both downstream signal transduction and enhancer and chromatin activation and accessibility, facilitating tumorigenesis and tumor plasticity (Figure 2).

### 3.5. Osteosarcoma

Cell culture studies have revealed that activation of the JNK branch of the MAPK pathway contributes to osteoblast differentiation and plays an important functional role in osteosarcoma development [74]. Immunohistochemical analyses revealed activation and expression of JNK/AP-1 signaling pathway signaling components in osteosarcoma tissues, but not in normal bone, with markedly higher levels observed in high-grade relative to low-grade tumors, suggesting a role in tumor progression [74]. Furthermore, while JNK and c-Jun were present in both the cytoplasm and nucleus of osteosarcoma cells, their activated/phosphorylated forms (p-JNK and p-c-Jun), localized exclusively in the nucleus, where c-Jun dimerizes with c-Fos to form AP-1 complexes [75]. c-Fos itself is implicated in osteoblast differentiation and osteosarcoma progression through its transcriptional upregulation via binding of the JNK-mediated phosphorylated Elk-1 to the serum response element [75,76]. A correlation between N-acetylcysteine (NAC) and c-Jun/p-c-Jun expression levels suggests dominance of c-Jun homodimers in active AP-1 complexes in human osteosarcomas. In conclusion, high immunoexpression of c-Jun and p-c-Jun predict high tumor grade, whereas c-Jun appears to be a more significant predictor than p-c-Jun [75]. The JNK/AP-1 signaling axis likely cooperates with Runt-related transcription factor 2 (RUNX2), a master regulator of osteoblast differentiation and growth, whose expression is markedly elevated in osteosarcomas [75,77].

### 3.6. Melanoma

Hyperactivation of MAPK signaling, a hallmark of *BRAF*-mutant melanomas, influences the therapeutic efficacy of MAPK inhibition. However, cellular heterogeneity in differentiation states leads to various responses to MAPK inhibitors both across genetically homogeneous populations of cells and across tumors of different genetic backgrounds [78]. Single-cell analyses conducted by Comandante-Lou et al. showed that the balance among AP-1 family members determines melanoma’s different phenotypes. Specifically, c-Fos and phosphorylated c-Fos (p-c-Fos) are linked to melanocytic states, whereas Fra-1, Fra-2, and c-Jun associate with less differentiated phenotype [79]. Furthermore, disruption of AP-1 components either by siRNA knockdown or MAPK pathway inhibition promotes transition between differentiation states and increased cellular heterogeneity [80].

Consistent with these findings, functional genomic studies demonstrated that c-Jun antagonizes the melanocytic lineage transcription factor MITF, which favors melanoma dedifferentiation and the acquisition of a pro-inflammatory phenotype [81]. AP-1 acts as a mediator switching the transcription potential of melanoma cells from a melanocytic program to inflammatory. This reciprocal c-Jun/MITF interconnection to transcriptional reprogramming and phenotypic plasticity, is associated with reduced sensitivity to MAPK-targeted therapies and may enable therapeutic resistance [81] (Figure 2).

### 3.7. Pancreatic Cancer

The AP-1 repressor Jun dimerization protein 2 (JDP2) is frequently downregulated in pancreatic cancer, and its decreased expression has been correlated with tumor invasion, metastasis, and poor patient survival. These observations suggest that JDP2 may function as a potential prognostic marker and contribute to the pathogenesis of pancreatic cancer [82]. Furthermore, Jun and Fos family proteins, together with ATF2, are expressed in pancreatic cancer cells, while c-Jun and ATF2 undergo phosphorylation on specific sites regulated by JNK, at Ser63 and Thr71 respectively [83].

Apurinic/apyrimidinic endonuclease 1/redox effector factor 1 (APE1/Ref-1), a redox signaling protein, is upregulated in pancreatic cancer and promotes tumor growth while its inhibition suppresses AP-1 activity and impairs carcinogenesis [84,85]. Additionally, the transcriptional co-activator YAP cooperates with AP-1 to promote the initiation of pancreatic tumorigenesis. AP-1 inhibition induces cell death in YAP-activated organoids, blocking pancreatic tumorigenesis in vivo, revealing a functional interaction between AP-1 and Hippo signaling [86]. Mechanistically, the activation of the AP-1 complex is induced by RAS-driven oncogenic signaling pathways, which lead to the invasion of pancreatic tumors [82]. Those pathways usually induce *Fra1*, *Fra2*, *c-Jun*, and *JunB*, but not c-Fos or FosB. Importantly, RAS transformation cannot occur in the absence of c-Jun. In conclusion, given the high mutation rates of *KRAS* in pancreatic cancer, AP-1 is considered a critical downstream effector and an exploitable treatment target [87].

AP-1 also cooperates with the nuclear effectors of the Hippo pathway YAP/TAZ and TEAD at specific enhancer elements. These transcription factors co-occupy regulatory regions to activate genes involved in proliferation, survival and tumor progression. This enhancer-centered mechanism further supports the role of AP-1 in pancreatic cancer, where it integrates oncogenic signals. In addition, various genetic changes and diseases have been related by high-throughput genomic investigations [26]. PerturbFate is a high-throughput, economical, epigenomic, combinatorial-indexing single-cell platform that links multimodal regulatory dynamics to disease-relevant phenotypes. It further confirmed that melanoma dedifferentiation is accompanied by increased chromatin accessibility at AP-1 family transcription factor binding regions, including JUN, FOSL1 and FOSL2, together with TEAD motifs [26,88].

### 3.8. Prostate Cancer

In contrast to its oncogenic role in pancreatic cancer, in prostate cancer, AP-1 may exhibit anti-tumor effects. For example, Fos protein demonstrates anti-tumor activity, whereas reduced expression of JunB and elements of the JNK pathway have been observed in advanced disease [89]. The appropriate function of AP-1 activity depends on a precise balance between its components to regulate transcription, and any disruption of this can lead to varying phenotypes. Notably, the loss of JunB or Fos together with phosphatase and tensin homolog (PTEN) deficiency can drive prostate cancer progression to the invasion stage, whereas c-Jun can act as an oncogene in the absence of Fos [89]. In addition to expression, chromatin remodeling also contributes to AP-1 function in prostate cancer. Recent studies have implicated FOXA2 in prostate cancer lineage plasticity, driving relocation of Jun chromatin-binding sites toward lineage-specific development enhancers, thereby promoting transcriptional reprogramming [90] (Figure 2).

### 3.9. Liver Cancer

Similarly, AP-1 activity contributes to liver tumorigenesis through distinct molecular pathways. A recent study showed that hepatic expression of c-Jun/Fra-2 promotes hepatocyte proliferation, reduces hepatic fat content, inducing liver inflammation and fibrosis. Subsequently, these changes lead to the development of liver tumors that have hepatocellular carcinoma characteristics [91]. In the tumors that evade growth suppression by switching off the c-Jun/Fra-2 AP-1 complex, alternative signaling routes may sustain MYC expression. For example, activation of the interleukine-6 (IL-6)/Janus kinase (JAK)/signal transducer and activator of transcription 3 (STAT3) pathway enhances *MYC* transcription, while PI3K/AKT–mediated inhibition of GSK3β stabilizes MYC protein. These mechanisms, together with increased Fos expression observed in some tumors, may restore AP-1-like transcriptional activity and support continued tumor proliferation [91].

Further to the promotion of hepatocyte proliferation, AP-1 regulates enhancer presence related to inflammation and fibrosis. Continuous AP-1 activation promotes chromatin remodeling, which facilitates the shift from chronic inflammation to hepatocellular carcinoma. This transition emphasizes its role in epigenetic adaptation during liver tumorigenesis [73] (Figure 2).

### 3.10. Glioblastoma

Glioblastoma (GBM) is a highly heterogeneous tumor, highly resistant to therapy and often recurrent. Interestingly, AP-1 has emerged as a transcriptional regulator of GBM heterogeneity and invasion. Studies using integrated single-nucleus RNA-seq and ATAC-seq analyses in matched tumor core (TC) and peritumoral brain (PTB) samples revealed that AP-1 activity is maintained across GBM cell states but decreases from the TC to the PTB, while the oxidative stress regulating transcription factor BTB and CNC homology 1 (BACH1) displays an opposite expression pattern [92]. In this context, combined inhibition of AP-1 and BACH1 can target GBM heterogeneity effectively [92].

Further studies have focused on controlling the functional molecular system that guides GBM proliferation and recurrence, aiming at maintaining GBM stem and precursor cells (GPCs) in an undifferentiated state [93]. In genetically modified-patient GPCs the transcription factor SRY-box transcription factor 21 (SOX21) has been shown to effectively suppress the tumorigenic potential, leading to improved patient survival. Mechanistically, it was shown that SOX21 acts by epigenetically suppressing AP-1-activated genes through binding to AP-1-targeted chromatin regions [93]. Consistent with this, pharmacological inhibition of AP-1 decreases GPC proliferation and survival, whereas overexpression of c-Jun, oppose these effects. Overall, SOX21-regulated pathways can suppress GPC malignancy by modulating AP-1 transcriptional programs, placing this pathway as a potential therapeutic target for GBM [93] (Figure 2).

### 3.11. Non-Malignant Pathologies

Even though the AP-1 pathway’s key molecules have been extensively studied in malignancies, evidence from non-malignant pathologies highlights its role as a stress-responsive transcription factor. In polycystic ovary (PCO) tissue, the upregulation of ERK1/2 and c-Jun in the follicular cell layers has been observed and this was accompanied by activation of AP-1 and nuclear factor-κB (NF-κB), transcription factors [94]. Similarly, advanced glycation end-products (AGEs) induce lysyl oxidase (LOX) and endothelin-1 (ET-1) overexpression in arterial endothelial cells, via ERK1/2 and JNK signaling and subsequent specific AP-1 and NF-κB activation [95,96]. DNA-protein binding assays further show that AP-1/p-c-Jun interacts with promoter regions of *LOX* and *ET-1* [94,97]. Although these results are obtained from non-tumor models, they resemble mechanisms seen in oncogenesis, such as chronic inflammation, oxidative stress, redox imbalance and endothelial dysfunction.

It can be seen that AP-1 biological function is highly dependent on cellular context, tumor type and subunit structure. AP-1 family members and their function across different cancer types are summarized in Table 1.

## 4. AP-1 in Immune Responses and the Tumor Microenvironment

**Figure 3:**
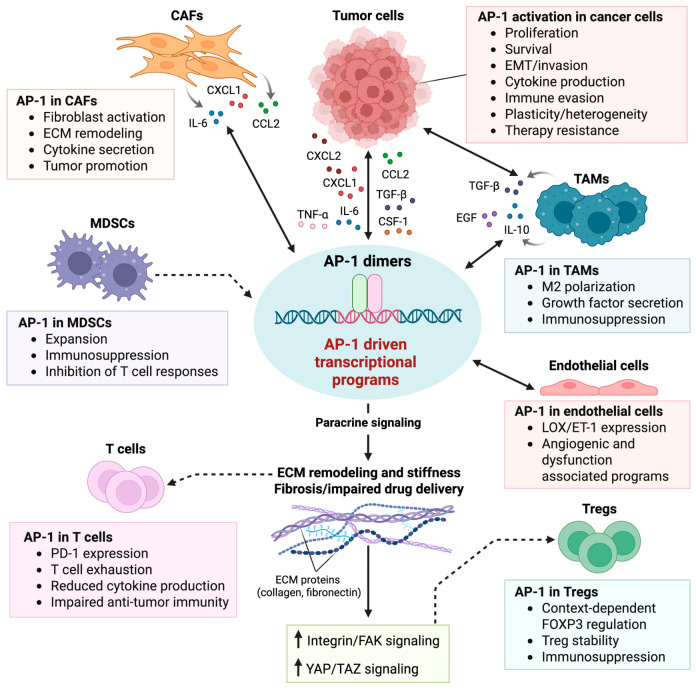
AP-1-driven transcriptional programs shape the tumor microenvironment. AP-1 activity regulates tumor–stroma–immune communication by controlling transcriptional programs in cancer cells and multiple components of the TME, including CAFs, TAMs, MDSCs, T cells, Tregs, and endothelial cells. In cancer cells, AP-1 supports proliferation, survival, EMT/invasion, cytokine production, immune evasion, plasticity/heterogeneity, and therapy resistance. In the TME, AP-1-associated programs contribute to CAF activation, ECM remodeling, myeloid-cell immunosuppression, T cell dysfunction, Treg stability, and endothelial angiogenic or dysfunction-associated states. AP-1-regulated mediators, including IL-6, TNF-α, CXCL1, CXCL2, CCL2, TGF-β, CSF-1, EGF, and IL-10, reinforce paracrine signaling, fibrosis, ECM stiffness, impaired drug delivery, and integrin/FAK–YAP/TAZ signaling. AP-1, activator protein-1; CAFs, cancer-associated fibroblasts; CCL2, C-C motif chemokine ligand 2; CSF-1, colony-stimulating factor 1; CXCL1/2, C-X-C motif chemokine ligand 1/2; ECM, extracellular matrix; EGF, epidermal growth factor; EMT, epithelial–mesenchymal transition; ET-1, endothelin-1; FAK, focal adhesion kinase; FOXP3, forkhead box P3; IL, interleukin; LOX, lysyl oxidase; MDSCs, myeloid-derived suppressor cells; PD-1, programmed cell death protein 1; TAMs, tumor-associated macrophages; TAZ, transcriptional coactivator with PDZ-binding motif; TGF-β, transforming growth factor-β; TME, tumor microenvironment; TNF-α, tumor necrosis factor-α; Tregs, regulatory T cells; YAP, Yes-associated protein. Created in BioRender. Adamopoulos, C. (2026) https://BioRender.com/chubkal (accessed on 7 July 2026).

Apart from its direct role in cancer cells, AP-1 plays a critical role in shaping the tumor microenvironment (TME). AP-1 and particularly its Jun family members are involved in stromal reprogramming. In human breast cancer stroma, analyses of activated motifs of cancer-associated fibroblasts (CAFs) reveal elevated AP-1 binding sites, including Jun as well as increased levels of phosphorylated/activated levels of Jun in metastatic tumors compared to normal tissues (Figure 3) [99].

In pancreatic ductal adenocarcinoma, AP-1 regulates the expression of pro-inflammatory cytokines, including IL-6, TNF-α, as well as chemokine ligands 1/2 (CXCL1/2). This promotes the recruitment of immunosuppressive cells such as myeloid-derived suppressor cells (MDSCs) and tumor-associated macrophages (TAMs) [98]. These cells secrete growth factors and matrix-remodeling enzymes that support tumor progression, while TAMs contribute to metastasis by developing a pro-tumorigenic M2 phenotype [100]. Thus, in this process, immunosuppressive conditions favor the development of the TME [101]. Single-cell transcriptomics in PDAC demonstrate that CAFs depict heterogeneity and diversity, consisting of multiple subtypes with variable clinical aggressiveness and clinical relevance [102]. Some subtypes of CAFs show poorer patient prognosis. It is revealed that the plasticity of CAFs responsible for their transition from normal fibroblasts to tumor-promoting phenotypes is controlled by FOS and JUN, thus reprogramming normal fibroblasts into CAFs [102]. The plasticity of CAFs is triggered by cytokine signaling within the TME, particularly via TGF-β and IFN-γ [102,103]. Additionally, the activation of AP-1 plays a role in the activation of pancreatic stellate cell (PSC) and fibrosis, which supports tumor growth and restricts drug delivery and treatment (Figure 3) [104,105].

Interestingly, AP-1 interacts with transcriptional co-activators of the Hippo pathway, such as YAP/TAZ, and regulates, via enhancers, inflammatory and tumor-promoting genes in the TME [26]. Beyond biochemical signaling, mechanical cues within TME regulate AP-1 activity. The increased stiffness of the extracellular matrix (ECM) caused by fibrosis results in the activation of integrin/focal adhesion kinase (FAK) signaling and the Hippo pathway effectors like YAP/TAZ, which bind AP-1 on enhancers, and promote tumorigenesis and inflammation (Figure 1 and Figure 3) [26,106].

AP-1 also mediates immune evasion. In immune cells, AP-1 shares its pioneer transcription features by establishing enhancer landscapes regulating inflammatory gene expression [4]. By regulating the transcription of immune checkpoint molecules such as programmed death-ligand 1 (PD-L1) and programmed cell death protein 1 (PD-1), AP-1 subunits (e.g., c-Jun, JunB, and c-Fos) directly contribute to the suppression of anti-tumor immunity in several solid tumors. In lung cancer and Epstein–Barr virus (EBV)-associated malignancies such as Hodgkin lymphoma and nasopharyngeal carcinoma, c-Jun and JunB were found to bind the PD-L1 promoter and induce PD-L1 expression in a MAPK-dependent manner [107,108]. In addition, interaction of the c-Fos complex with c-Jun and JunB at the PD-1 promoter enhances PD-1 expression in tumor-infiltrating T cells, suppressing anti-tumor immune responses [109]. In chronic viral infections, elevated PD-1 expression inhibits proliferation and cytokine production, leading to T cell exhaustion through upregulation of BATF, a member of the AP-1/ATF transcription factor subfamily [98]. Finally, cytotoxic T-lymphocyte-associated protein 4 (CTLA-4) ligation studies have been shown to disrupt CD28/T-cell receptor (TCR)-dependent signaling, especially ERK- and JNK-mediated MAPK activation, contributing to immune checkpoint inhibition through reduced AP-1 DNA binding activity (Figure 3) [110].

Recent research further illustrated that the AP-1 subunit c-Jun directly regulates PD-L1 expression in drug-resistant cancer cells. Silencing c-Jun reduced PD-L1 expression notably, whereas c-Jun overexpression enhanced PD-L1 levels. This suggests that AP-1 contributes to immune evasion and may influence response to the blockage of immune checkpoints [111]. Consequently, therapeutic strategies combining AP-1 inhibition with PD-1/PD-L1 blockade may pose an effective strategy in overcoming immunotherapy resistance.

In regulatory T cells (Tregs), AP-1 proteins directly interact with regulatory regions of the *forkhead box P3* (*Foxp3*) promoter and facilitate its transcriptional activation [112]. In addition, TCR-TGF-β signaling induces Foxp3 activation in a MAPK-dependent manner via AP-1 activation [113]. Conversely, induction of BATF-3 complex following the co-stimulatory receptor OX40 ligation in Tregs, negatively regulates *Foxp3* transcription by binding to its promoter and suppressing its expression (Figure 3) [114].

## 5. Inhibition of AP-1 and Therapies

AP-1 has emerged as an attractive therapeutic target due to its diverse and strong oncogenic impact. Current therapeutic strategies focus on three main approaches: (a) direct inhibition, by direct dysregulation of the AP-1 binding domain or dimerization, (b) modulation of upstream pathways that regulate AP-1 expression, (c) targeting of AP-1 TRE DNA binding sites, its transcriptional co-activators and protein–protein interactions.

### 5.1. Direct or Indirect Inhibition by Small Molecule Inhibitors

A small molecule c-Fos/AP-1 inhibitor (T-5224) directly inhibits AP-1 binding to the DNA and has shown efficacy in preclinical models of inflammatory disease [115]. Possible direct and indirect inhibitors of AP-1 transcription factor are summarized below (Table 2).

In head and neck squamous cell carcinoma (HNSCC), T-5224 suppresses invasion and migration, and reduces lymph node metastasis in an orthotropic tumor implantation mouse model, leaving the tumor unaffected; however, its effect on primary tumor growth appears limited [116]. These effects may be mediated through a potential interplay with other factors such as IL-1, IL-6, TNF-α, matrix metalloproteinases (MMPs) and others [115,116]. Another compound that targets AP-1 function, a topoisomerase inhibitor, XR5944 or MLN944, possesses anti-tumor activity in vitro and in vivo. Mechanistically, XR5944 is a DNA bis-intercalator that prevents c-Jun from binding to AP-1 TRE sequences, blocking its transcriptional activity. At a preclinical level, XR5944 delayed tumor growth in colon carcinoma models [117]. Similarly, an AP-1 transcriptional activity inhibitor, a synthetic retinoid, SR11302 suppresses Fra-1/AP-1 binding to TRE sites, reducing tumor growth and lymph node metastasis in HNSCC models [127]. Another compound, a dual AP-1/NF-κB transcriptional activation inhibitor, SPC839, suppresses AP-1 activity and exhibits inhibitory effects on nitric oxide, TNF-α and the receptor tyrosine kinase FLT3, which are potential therapeutic targets for acute myeloid leukemia (AML) [118]. Finally, the reversible, ATP-competitive small molecule JNK inhibitor SP600125 can inhibit AP-1 activity [122].

### 5.2. Natural and Plant-Derived Compounds

Several natural agents with potent anti-AP-1 activity have been identified including anthocyanins, which inhibit both the DNA-binding activity and nuclear translocation of AP-1 proteins [123]. Veratramine, a steroidal alkaloid isolated from *Veratrum* species, binds to specific TRE within AP-1 target genes and regulates AP-1-dependent transcription [124]. Also, curcumin, a polyphenolic compound from turmeric, and its synthetic derivatives seem to suppress the formation of DNA-Jun-Fos complexes. Interestingly, combination therapies with natural compounds are often more effective. Phytochemicals and kinase inhibitors exhibit cumulative effects in combating tumorigenesis [125]. AP-1 can be inhibited by blocking the activity of upstream effector molecules using natural or synthetic agents. For example, ascochlorin and silibinin, target ERK1/2, while flavonoids, such as kaempferol and genistein, inhibit both JNK and ERK1/2, and finally resveratrol, which targets MEK1 [130].

Despite these promising preclinical findings, the clinical translation of natural AP-1 inhibitors remains challenging. The limitations that should be addressed are poor bioavailability, limited target specificity, and variable pharmacokinetic identities [131]. Therefore, strategies such as analytical tools, structural sequencing, and combination therapies are being explored to enhance therapeutic potential and facilitate clinical application.

### 5.3. Targeting AP-1’s Upstream Regulators or Downstream Targets

Inhibition of AP-1 by upstream targeting of the MAPK/ERK pathway has been shown in various cases. *BRAF*-mutant melanoma cells exhibit cell pattern differentiation when treated with the BRAF inhibitor vemurafenib either alone or in combination with a MEK inhibitor, resulting in potent suppression of the ERK1/2 phosphorylation and AP-1 function [79]. Furthermore, c-Jun and p-c-Jun expression changes can predict both drug-induced dedifferentiation and differentiation in melanoma. Elevated c-Jun and p-c-Jun levels have been observed in undifferentiating melanoma cells, while reduced expression of these proteins characterizes differentiating melanoma cells following MAPK inhibitor treatment [120,132]. Melanoma cell lines that were treated with the combination of vemurafenib and trametinib displayed significantly lower levels of ERK phosphorylation, while phosphorylated-Fra1 emerged as the most consistent predictor of ERK pathway inhibition efficacy among various cell lines. In addition, pharmacological inhibition of ERK signaling may lead to AP-1 signaling rewiring [133].

Another approach of targeting downstream transcriptional targets of AP-1 could be promising. For example, although frontline treatment of large cell lymphoma, ALK-positive ALCL, relies on combination chemotherapy regimens, therapies targeting AP-1 transcriptional downstream effectors have shown clinical efficacy. Thus, inhibition of PDGFRβ, which is transcriptionally activated by Jun and JunB, with the tyrosine kinase inhibitor imatinib in an advanced ALK-positive ALCL patient resulted in a durable response Moreover, galectin-1, a β-galactoside-binding lectin, is associated with a CRAF-mediated AP-1 activation and its knockdown improved sensitivity to chemotherapy drugs in breast cancer cell lines [121,134].

### 5.4. Epigenetic and Chromatin Modifiers

Epigenetic modulators also can influence AP-1 activity. Resminostat, a histone deacetylase (HDAC) inhibitor, can occupy AP-1 DNA binding sites and affects c-Jun by increasing acetylation of both c-Jun and histones and modulating AP-1 target gene transcription [135]. In this context, the combination of resminostat and the multikinase inhibitor, sorafenib, can act protectively in hepatocellular carcinomas, suppressing the MAPK/ERK signaling pathway [135]. Other HDAC inhibitors (e.g., SAHA, TSA, Panobinostat) similarly alter AP-1 activity, although their effects are tumor type dependent and their effectiveness is higher when used in combination with anti-tumor drugs or radiotherapy [126,135].

Activated AP-1 itself appears to promote the interaction between promoters and enhancers, inducing chromatin looping at active transcription sites in NSCLC. Interestingly, inhibition of AP-1 or of JNK upstream suppresses oncogenes, interfering with these epigenetic interactions [70].

### 5.5. Emerging Therapy Directions

Disruption of dimerization of the AP-1 monomeric components and the subsequent inhibition of AP-1 complex formation represent a novel therapeutic approach for targeting AP-1-driven tumors. JunAP is a peptide antagonist that selectively targets AP-1 family members, such as Fra-1 and Jun, disrupting their interactions and blocking AP-1 transcriptional activity. In parallel, oligonucleotides (ONTs), including antisense oligonucleotides, small-interfering RNAs (siRNAs), self-amplifying RNAs (saRNAs), microRNAs (miRNAs), decoys and aptamers, present an evolving cancer treatment field, due to their specificity and capacity to regulate gene transcription. An AP-1 targeting decoy oligodeoxynucleotide has been used in the past to block intestinal inflammation in a mouse model of experimental colitis [128,136].

Another promising therapeutic strategy involves RNA interference (RNAi) and CRISPR-based silencing of key oncogenic components of AP-1 complex, including Fra-1 and Jun [87,137,138]. TNBC-multiomic profiling has identified Fra-1-induced distinct super-enhancer patterns and TNBC-specific super-enhancers, such as the treacle ribosome biogenesis factor 1 (TCOF1), associated with oncogenesis. In particular, Fra-1 binds to a super-enhancer of TCOF1 further activating its transcription. Hence, Fra-1 appears to have a key epigenetic role and targeting of the Fra-1-TCOF1 axis may pose a potential therapeutic strategy for TNBC [139,140].

Finally, proteolysis targeting chimeras (PROTACs), have been extensively tested preclinically to target and direct proteins of interest to degradation via ubiquitination, including several oncoproteins [141]. In this context, Fra-1 has been targeted by a new T-5224-based PROTAC in HNSCC, showing potential therapeutic applicability [129]. In addition, in spite of the encouraging findings, both PROTACs and oligonucleotides remain highly experimental. Obstacles that must be overcome include delivery and dosing, limited cellular uptake as well as pharmacokinetics that must be considered. Interestingly, therapeutic resistance can be influenced by changes in target proteins, E3 ubiquitin ligases or other intracellular mechanisms. Thus, to achieve an optimization of drug delivery, personalized therapeutic approaches could be an asset [142].

Despite the various preclinical findings, direct targeting of AP-1 remains challenging. The fact that AP-1 consists of a plethora of dimeric complexes whose functions vary depending on the cellular and tumor type, it is difficult to focus on a specific inhibition pattern. In addition, the structure of transcription factors does not facilitate the development of small-molecule effective inhibitors. Thus, current approaches aim at targeting upstream signaling pathways, AP-1 co-regulators, or alternative strategies such as RNA-based therapeutics and PROTACs [7,141].

## 6. Conclusions

AP-1 is a complex transcription factor highly dependent on context, which plays a major role in tumor progression, integrating oncogenic signaling pathways into transcription. AP-1 can control substantial cellular processes including proliferation, apoptosis, differentiation, invasion, EMT, and therapy resistance being regulated by MAPK, PI3K/Akt, inflammatory, stress-related, and epigenetic pathways. Interestingly, across various cancer types, AP-1 can trigger tumorigenesis by supporting transcriptional programs, chromatin remodeling, cancer cell plasticity, and metastatic behavior.

Notably, AP-1 is not only involved in tumor progression but also shapes the TME through regulation of inflammation, immune cell expression and interactions, stromal activation, and angiogenesis. However, AP-1 function can be defined by the tumor type, cellular context, component composition, and chromatin accessibility; hence, some members display tumor-suppressive effects in specific settings while others do not. Pharmacologically, the direct targeting of AP-1 remains challenging; however, recent preclinical studies support the effectiveness of direct inhibition, the blockage of upstream pathways, epigenetic modulation, and subsequent combination therapies. Future therapy includes disruption of AP-1 dimerization, RNA-based targeting, CRISPR-based silencing, and PROTAC development. Overall, AP-1 represents a promising therapeutic target despite its regulation complexity, a crucial interconnector of oncogenic pathways.

## Figures and Tables

**Table 1:** AP-1 family members and their function across different cancer types.

AP-1 Subunit	Cancer Type	Role	Function/Mechanism	References
c-Jun	Breast cancer, CRC, melanoma, liver cancer, ALCL	Oncogenic	Promotes proliferation, EMT, invasion, MYC activation, therapy resistance, PD-L1 expression	[9,91]
JunB	Breast cancer, prostate cancer	Tumor suppressive	Associated with lower tumor stage and reduced proliferation; context-dependent transcription	[34,89]
	ALCL, multiple myeloma, esophageal cancer	Oncogenic	Promotes proliferation, survival, PDGFRβ expression; poor prognosis	[7,16]
JunD	Breast cancer, CRC, prostate cancer	Tumor suppressive	Regulates differentiation and suppresses aggressive transcriptional programs	[3,34]
c-Fos	Lung cancer, breast cancer, osteosarcoma	Context-dependent	Cooperates with c-Jun during transformation; promotes differentiation in some tissues	[71,75]
	Prostate cancer	Tumor suppressive	Loss of c-Fos promotes progression together with PTEN deficiency	[89]
Fra-1 (FOSL1)	TNBC, CRC, lung cancer, pancreatic cancer	Oncogenic	EMT, invasion, stemness, enhancer remodeling, metastasis, therapy resistance	[36,44,51]
Fra-2 (FOSL2)	Breast cancer, liver cancer	Oncogenic	Cooperates with c-Jun; promotes proliferation, fibrosis and hepatocellular carcinoma	[91]
ATF2	Pancreatic cancer, melanoma	Context-dependent	Regulates stress responses, invasion and MAPK signaling	[79]
BATF	ALCL, immune cells	Context-dependent	Immune regulation, T-cell differentiation, AP-1 complex formation	[16,98]

ALCL, anaplastic large-cell lymphoma; AP-1, activator protein-1; ATF2, activating transcription factor 2; BATF, basic leucine zipper ATF-like transcription factor; CRC, colorectal cancer; EMT, epithelial–mesenchymal transition; FOSL1, Fos-like antigen 1/Fra-1; FOSL2, Fos-like antigen 2/Fra-2; MYC, MYC proto-oncogene/basic helix–loop–helix transcription factor; PD-L1, programmed death-ligand 1; PDGFRβ, platelet-derived growth factor receptor beta; PTEN, phosphatase and tensin homolog; TNBC, triple-negative breast cancer.

**Table 2:** Therapeutic strategies and key effects of targeting AP-1 signaling in cancer.

Strategy	Compound/ Approach	Target/ Mechanism	Cancer Type	Clinical Development	Key Effect	Ref.
Direct inhibition	T-5224	Blocks AP-1 (c-Fos/c-Jun) DNA binding	HNSCC	Phase II (rheumatoid arthritis); preclinical in cancer	↓ Invasion, ↓ metastasis	[116]
	MLN944	DNA bis-intercalator; prevents c-Jun binding to TRE	CRC	Preclinical	↓ Tumor growth	[117]
	SR11302	Inhibits Fra-1/AP-1 binding to TRE	HNSCC	Preclinical	↓ Tumor growth, ↓ metastasis	[116]
Indirect inhibition	SPC839	Suppresses NF-κB and AP-1 activity	AML	Preclinical	↓ NO, ↓ TNF-α, ↓ Proliferation	[118]
Direct/indirect inhibition	Pentraxin-2	Inhibits Jun/AP-1 signaling	ATL	Preclinical	↓ Cell growth	[119]
Upstream targeting	Vemurafenib + Trametinib	BRAF/MEK inhibition → ↓ ERK/AP-1	Melanoma	Approved therapy (FDA/EMA-approved for BRAF-mutant melanoma)	↓ Proliferation, ↑ differentiation	[120]
	Imatinib	Inhibits PDGFRβ (AP-1 downstream target)	ALK+ ALCL	Clinical use	Clinical response	[58]
	Galectin-1 inhibition	Disrupts Raf-1/ AP-1 signaling	Breast cancer	Preclinical	↑ Therapy sensitivity	[121]
	SP600125	JNK inhibitor → ↓ AP-1 activation	Multiple types	Preclinical	↓ Transcriptional activity	[122]
Natural compounds	Anthocyanins	Inhibit AP-1 DNA binding & nuclear translocation	Multiple types	Preclinical	↓ AP-1 activity	[123]
	Veratramine	Binds TRE sites; modulates AP-1 transcription	Multiple types	Preclinical	↓ AP-1 activity	[124]
	Curcumin	Disrupts Jun–Fos complex	Multiple types	Preclinical/Early clinical evaluation	↓ AP-1 activity	[125]
Epigenetic	Resminostat	HDAC inhibitor → ↑ c-Jun acetylation	HCC	Phase II (HCC and other solid tumors)	↓ ERK signaling, ↓ metastasis	[119]
	SAHA, TSA, Panobinostat	HDAC inhibition → chromatin remodeling	Multiple types	SAHA: Approved (for CTCL); preclinical in AP-1-targeting studies	AP-1 transcription modulation	[126]
Emerging	siRNA/ shRNA/ CRISPR	Silencing FOSL1, JUN	TNBC	Preclinical	↓ Oncogenic transcription	[127]
	Oligonucleotides (ASO, miRNA, aptamers)	Gene expression modulation	Multiple types	Preclinical/Early clinical development	↓ Metastasis, ↑ immunotherapy	[128]
	PROTACs	Targeted degradation of oncogenic proteins	Breast cancer, prostate cancers	Preclinical	↓ Tumor growth	[129]

ALK+ ALCL, anaplastic lymphoma kinase-positive anaplastic large cell lymphoma; AML, acute myeloid leukemia; AP-1, activator protein-1; ASO, antisense oligonucleotide; ATL, adult T-cell leukemia/lymphoma; BRAF, B-Raf proto-oncogene serine/threonine kinase; c-Fos, FBJ murine osteosarcoma viral oncogene homolog; c-Jun, Jun proto-oncogene, AP-1 transcription factor subunit; CRC, colorectal cancer; CRISPR, clustered regularly interspaced short palindromic repeats; DNA, deoxyribonucleic acid; ERK, extracellular signal-regulated kinase; FOSL1/Fra-1, FOS-like antigen 1; HDAC, histone deacetylase; HCC, hepatocellular carcinoma; HNSCC, head and neck squamous cell carcinoma; JNK, c-Jun N-terminal kinase; JUN, Jun proto-oncogene; MEK, mitogen-activated protein kinase kinase; miRNA, microRNA; NF-κB, nuclear factor kappa-light-chain-enhancer of activated B cells; NO, nitric oxide; PDGFRβ, platelet-derived growth factor receptor beta; PROTAC, proteolysis-targeting chimera; Raf-1, Raf-1 proto-oncogene serine/threonine kinase; SAHA, suberoylanilide hydroxamic acid/vorinostat; shRNA, short hairpin RNA; siRNA, small interfering RNA; TNBC, triple-negative breast cancer; TNF-α, tumor necrosis factor alpha; TRE, TPA-response element; TSA, trichostatin A; ↓, downregulation; ↑ upregulation; → induction.

## Data Availability

No new data were created or analyzed in this study. Data sharing is not applicable.

## Abbreviations

The following abbreviations are used in this manuscript:

ABC-DLBCL	Activated B-cell-like diffuse large B-cell lymphoma
AGEs	Advanced glycation end-products
ALCL	Anaplastic large cell lymphoma
ALK	Anaplastic lymphoma kinase
AML	Acute myeloid leukemia
AP-1	Activator protein-1
APE1/Ref-1	Apurinic/apyrimidinic endonuclease 1/redox effector factor
ASK1	Apoptosis signal-regulating kinase 1
ATAC-seq	Assay for Transposase-Accessible Chromatin sequencing
ATF	Activating transcription factor
BAF	BRG1/BRM-associated factor
BACH1	BTB and CNC homology 1
BATF	Basic leucine zipper ATF-like transcription factor
BRAF	B-Raf proto-oncogene serine/threonine kinase
bZIP	Basic region leucine zipper
cAMP	Cyclic adenosine monophosphate
CAFs	Cancer-associated fibroblasts
CARMA1	Caspase recruitment domain membrane-associated guanylate kinase
CBP	CREB-binding protein
ChIP-seq	Chromatin immunoprecipitation sequencing
cHL	Classical Hodgkin lymphoma
COX-2	Cyclooxygenase-2
CRC	Colorectal cancer
CRE	cAMP-responsive element
CRISPR	Clustered Regularly Interspaced Short Palindromic Repeats
CTLA-4	Cytotoxic T-lymphocyte-associated protein 4
CXCL1	C-X-C motif chemokine ligand 1
CXCL2	C-X-C motif chemokine ligand 2
E1A	Adenovirus early region 1A
EBV	Epstein–Barr virus
EGFR	Epidermal growth factor receptor
Elk-1	ETS Like-1 protein Elk-1
EMT	Epithelial–mesenchymal transition
ER	Estrogen receptor
ERK1/2	Extracellular signal-regulated kinase ½
ETS	ETS-domain transcription factor
ET-1	Endothelin-1
Fer-1	Ferroptosis-specific inhibitor ferropstatin-1
FoxO3	Forkhead box O3
Foxp3	Forkhead box P3
Fra-1	Fos-related antigen 1
GBM	Glioblastoma
GPCs	Glioblastoma precursor cells
GSK3β	Glycogen synthase kinase 3 beta
HDAC	Histone deacetylase
HNSCC	Head and neck squamous cell carcinoma
IFN-γ	Interferon gamma
ILC3	Group 3 innate lymphoid cells
IL-6	Interleukin-6
JAK	Janus kinase
JDP2	Jun dimerization protein 2
JNK	c-Jun N-terminal kinase
KRAS	Kirsten rat sarcoma viral oncogene homolog
LEF	Lymphoid enhancer factor
LOX	Lysyl oxidase
MAF	Musculoaponeurotic fibrosarcoma oncogene family
MAP2K	Mitogen-activated protein kinase kinase
MAP3K	Mitogen-activated protein kinase kinase kinase
MAPK	Mitogen-activated protein kinase
Mcl-1	Myeloid cell leukemia-1
MDSCs	Myeloid-derived suppressor cells
MEK1/2	Mitogen-activated protein kinase kinase 1/2
miRNA	MicroRNA
MM	Multiple myeloma
MMP	Matrix metalloproteinases
mTOR	Mammalian target of rapamycin
MyD88	Myeloid differentiation primary response gene 88
NAC	N-acetylcysteine
NF-κB	Nuclear factor kappa B
NSCLC	Non-small cell lung cancer
ONTs	Oligonucleotides
OX40	Tumor necrosis factor receptor superfamily member 4
p300	Binding protein p300
PCO	Polycystic ovary
PD-1	Programmed cell death protein-1
PDAC	Pancreatic ductal adenocarcinoma
PDGFRβ	Platelet-derived growth factor receptor beta
PD-L1	Programmed death-ligand 1
PI3K	Phosphatidylinositol 3-kinase
PKC	Protein kinase C
PROTAC	Proteolysis targeting chimera
PSC	Pancreatic stellate cell
PTB	Peritumoral brain
PTF	Pioneer transcription factor
PTEN	Phosphatase and tensin homolog
RARα	Retinoic acid receptor alpha
RAS	Rat sarcoma
RNAi	RNA interference
RTK	Receptor tyrosine kinase
RUNX2	Runt-related transcription factor 2
saRNA	Self-amplifying RNA
SEK1	Stress-activated protein kinase/ERK kinase 1
SHH	Sonic Hedgehog
shRNA	Short hairpin RNA
siRNA	Small interfering RNA
SOX21	SRY-box transcription factor 21
STAT3	Signal transducer and activator of transcription 3
SWI/SNF	Switch/sucrose non-fermentable
TAMs	Tumor-associated macrophages
TAZ	Transcriptional co-activator with PDZ-binding motif
TC	Tumor core
TCF	Ternary complex factor/T-cell factor
TCOF1	Treacle ribosome biogenesis factor 1
TCR	T-cell receptor
TGF-β	Transforming growth factor beta
TH17	T helper 17 cells
TME	Tumor microenvironment
TNBC	Triple-negative breast cancer
TNF-α	Tumor necrosis factor alpha
TPA	12-O-tetradecanoylphorbol-13-acetate
Tregs	Regulatory T cells
TRE	TPA-response element
USP21	Ubiquitin-specific protease 21
VEGF	Vascular endothelial growth factor
YAP	Yes-associated protein
ZEB1	Zinc finger E-box binding homeobox 1
ZEB2	Zinc finger E-box binding homeobox 2
